# Latent profiles of exercise motivation and exercise-induced emotions: associations with physical activity and gender among Chinese college students

**DOI:** 10.3389/fpsyg.2026.1759866

**Published:** 2026-03-05

**Authors:** Yueqiang Dai, Ying Zhao, Pan He

**Affiliations:** 1 Minnan Science and Technology College, Nanan, China; 2 Sports Science and Technology, Bangkok Thonburi University, Bangkok, Thailand; 3 School of Physical Education and Health, Sanming University, Sanming, China; 4 College of Humanities, Yiyang Medical College, Yiyang, China

**Keywords:** exercise-induced emotions, gender differences, latent profile analysis, motivation, physical activity

## Abstract

Although the specific reasons for exercise motivation and the emotions felt during it are both important for physical activity (PA), little is known about how they combine to form distinct psychological profiles. This study used a person-centered approach to identify these latent profiles based on specific exercise motives and exercise-induced emotions among Chinese college students, and examined their associations with gender and PA. We recruited 1,586 undergraduates from a university in southern China (*M_age_* = 19.13, *SD* = 1.23; 468 males, 1,118 females). They completed the Motives for Physical Activities Measure-Revised (MPAM-R), the Exercise-Induced Feeling Inventory (EFI), and the International Physical Activity Questionnaire-Short Form (IPAQ-SF). Latent profile analysis (LPA) was conducted to identify subgroups based on their motivational and emotional patterns. The results supported a three-profile model: Low-Enjoyment and High-Exhaustion Profile (14.7%), Appearance-Driven and Ambivalent-Affect Profile (50.6%), and Enjoyment-Driven and High-Vitality Profile (34.8%). Gender significantly predicted profile membership, with male students more likely to belong to the Enjoyment-Driven and High-Vitality Profile. Physical activity levels differed significantly across the latent profiles in a specific pattern. The Enjoyment-Driven and High-Vitality Profile demonstrated a higher level of physical activity than both the Low-Enjoyment and High-Exhaustion Profile and the Appearance-Driven and Ambivalent-Affect Profile. No significant difference in physical activity was found between the latter two profiles. These findings reveal three distinct experiential patterns and highlight the large subgroup driven by appearance concerns as a key target for interventions aimed at fostering more autonomous motivation and positive affective experiences.

## Introduction

Physical inactivity among college students has become a major global public health issue affecting countries across all income levels (Jiang et al., 2024; Pengpid et al., 2015; Uddin et al., 2020). This low activity level poses significant health risks for college students, including higher odds of obesity, increased symptoms of anxiety and depression, and reduced academic performance (Castro et al., 2018, 2020). In contrast, regular physical activity (PA) offers clear benefits, which support physical health and enhance psychological well-being by boosting positive emotions and lowering stress in young adults (Ashford et al., 2010; Pengpid et al., 2015). Given these impacts, researchers and public health professionals worldwide have prioritized understanding the factors that drive or hinder college students’ physical activity.

Despite growing attention to this area, existing studies on exercise-related psychology in college populations have key gaps, particularly in their methodological approaches. Most prior research relies on variable-centered methods, which focus on individual factors such as exercise motivation or exercise-induced emotions in isolation rather than exploring how these factors interact. Very few studies, however, have used person-centered methods to explore how motivation and emotion combine to form distinct psychological profiles among students (Rose and Parfitt, 2007). While recent person-centered research has begun to profile motivation or examine its links to behavior across different contexts (Zhao et al., 2025), studies that integrate specific exercise motives with exercise-induced emotions remain scarce. This is a critical limitation because person-centered approaches are better suited to capturing the heterogeneity of students’ exercise experiences, revealing the difference of motivation alongside emotion (Ferguson et al., 2020; Spurk et al., 2020). Additionally, while gender and age are known to influence exercise motivation and emotions, few studies have systematically tested how these demographic factors shape the formation of exercise-related psychological profiles.

The purposes of this study are to address these gaps by identifying latent profiles of exercise motivation and exercise-induced emotions, examining whether gender and age predict membership in these profiles, and exploring differences in PA across the identified profiles. This study will contribute to theoretical advancements in exercise psychology and support the development of targeted interventions for college students, aligning with global efforts to improve young adults’ physical activity.

### Physical inactivity in the college student

The transition to university life represents a critical period for the establishment of long-term health behaviors. Despite the well-documented benefits of regular physical activity (PA), a substantial body of evidence indicates that college students across the globe exhibit disturbingly high rates of physical inactivity (Buecker et al., 2021; Hong et al., 2020). This phenomenon is not confined to any single region but constitutes a widespread public health concern affecting diverse cultural and economic contexts (Pengpid et al., 2015; Uddin et al., 2020).

In China, the situation is particularly noteworthy. A combination of intense academic pressures, competitive environments, and increased sedentary behaviors associated with university coursework contributes to a significant decline in PA participation during these years (Hong et al., 2020; Jiang et al., 2024). This low level of activity is associated with a range of immediate and long-term adverse outcomes, including elevated risks of obesity, metabolic disorders, and diminished psychological well-being, such as increased symptoms of anxiety and depression (Castro et al., 2020).

This pervasive issue underscores the urgent need to move beyond merely documenting low activity levels and toward a deeper understanding of the underlying psychological mechanisms that drive or hinder exercise behavior. Identifying these mechanisms is a prerequisite for designing effective, theory-based interventions aimed at reversing the trend of physical inactivity among this vulnerable demographic group.

### From regulatory styles to motives: bridging theory and measurement

Within the framework of Self-Determination Theory (SDT), motivation in the context of exercise is conceptualized as a continuum, ranging from amotivation to intrinsic motivation (Deci and Ryan, 2012; Teixeira et al., 2012). Building on the foundational SDT principle that the quality of motivation is paramount, this study examines the specific content of exercise motivation. While SDT delineates a continuum of regulatory styles (e.g., intrinsic, identified, introjected), assessing these styles directly requires instruments focused on the perceived locus of causality. For the applied purpose of understanding the substantive reasons students engage in exercise, we instead employ the Motives for Physical Activities Measure–Revised (MPAM-R) (Frederick and Ryan, 1993). The MPAM-R assesses five key motive domains: enjoyment, competence, appearance, fitness, and social interaction.

Critically, these specific motivational contents are not theoretically independent of SDT. A robust body of research establishes a systematic conceptual and empirical link between motive domains and the broader regulatory continuum. Motives oriented toward inherent interest (enjoyment) and mastery (competence) are consistently associated with more autonomous or self-determined forms of regulation. In contrast, motives focused primarily on appearance—often driven by social comparisons or external approval—typically correlate with more controlled forms of motivation, such as introjected or external regulation (Teixeira et al., 2012; Ryan and Deci, 2000). Fitness and social motives can exhibit more variability, potentially aligning with either autonomous or controlled regulation depending on their personal integration. Therefore, profiling individuals based on their configuration of these specific motives provides a valid and practical means of capturing meaningful variance in their underlying motivational orientation, as conceptualized by SDT.

This person-centered investigation of motive profiles, when integrated with patterns of exercise-induced emotions, allows for a holistic analysis. It enables the identification of how practically reported reasons for exercise coalesce with affective experiences to form distinct psychological subgroups, addressing the central aim of this study.

### Empirical research on exercise motivation, emotions, and physical activity

Guided by the understanding that motivational quality is central, empirical research has extensively examined the links between motivation, affective experiences, and physical activity behavior. The evidence consistently shows that these psychological factors are dynamically interconnected, influencing exercise participation and adherence.

The most robust finding is the direct link between the emotions and sustained behavior. Studies confirm that when individuals feel positive emotion and competent in their exercise, they are more likely to maintain regular physical activity over time (Fortier et al., 2007; Teixeira et al., 2012). Conversely, exercising primarily due to external pressure or to avoid guilt is consistently associated with lower participation and higher dropout rates (Gillison et al., 2019).

Beyond direct effects, affective responses serve as a critical mechanism. Engaging in exercise for self-determined reasons tends to satisfy basic psychological needs, which fosters positive emotions such as vitality and enjoyment (Ryan and Sapp, 2007). Research supports this: individuals who exercise for autonomous reasons report more positive affect, while those driven by controlled motives report more negative affect, including tension and exhaustion (Rose and Parfitt, 2007). These emotional experiences are not just outcomes; they create a feedback loop. The Affect-Health Framework suggests people are motivated to repeat activities that feel good and avoid those that feel bad (Williams et al., 2018). Therefore, positive exercise emotions can reinforce motivation and future participation, while negative emotions can undermine motivation and lead to discontinuation (Rhodes and Kates, 2015).

Applied to the study of specific exercise motives, this body of work suggests that motives linked to self-determination (e.g., enjoyment, competence) should coincide with more positive emotional experiences and better behavioral outcomes. In contrast, motives reflecting external pressures (e.g., appearance concerns driven by social standards) may coincide with more conflicted or negative affective states (Frederick and Ryan, 1993).

The literature thus reveals a complex interplay where motivation influences behavior both directly and indirectly through emotion. However, most evidence comes from variable-centered studies examining these relationships in isolation. This approach cannot reveal how specific combinations of motivational contents and emotional experiences naturally cluster within individuals. A more holistic, person-centered investigation is needed to understand these real-world psychological configurations (Spurk et al., 2020).

### Gender and age differences in physical activity

A comprehensive understanding of physical activity behaviors necessitates the consideration of key demographic variables, among which gender and age represent two of the most widely examined factors.

Research consistently shows significant gender disparities in physical activity among college students. Male students typically report higher overall activity levels, engage in more vigorous exercise, and report stronger motivation for exercise compared to female students (Chalabaev et al., 2013; Riemer and Visio, 2003). These differences are linked to socio-cultural factors such as gender socialization, differing perceptions of competence, and specific barriers. For example, female students may experience greater appearance-related anxiety, lower self-efficacy in certain sports, or safety concerns that hinder regular participation (Boiché et al., 2014; Saemi et al., 2023). In the specific context of Chinese higher education, these patterns may be further accentuated by traditional norms, making gender a critical variable for profiling exercise psychology.

Extending beyond activity levels, gender differences are also observed in the specific reasons for exercise. Studies suggest that males often score higher on motives related to competition and strength, while females may report greater emphasis on motives related to appearance and weight management (Frederick and Ryan, 1993). This indicates that gender may shape not only how much individuals exercise, but also their underlying motivational profile.

The relationship between age and physical activity during young adulthood is less clear. The college years are often marked by a decline in structured activity as students face new academic and social demands (Kwan et al., 2012). Evidence on age-related changes within the undergraduate years is inconsistent, with some studies noting a slight decrease in activity and others finding no significant change (Chalabaev et al., 2013). This inconsistency highlights the value of examining age as a potential factor in shaping exercise psychology.

Critically, while gender and age are known correlates of physical activity volume, their role in shaping integrated psychological configurations is less explored. Most research examines these demographics in relation to isolated variables. It remains unclear whether and how gender and age predict an individual’s likelihood of belonging to a particular holistic profile characterized by a specific combination of exercise motives and emotions. Understanding this can provide a stronger foundation for developing targeted physical activity interventions for specific subgroups.

### The person-centered approach and current gaps

Despite the valuable insights provided by the aforementioned variable-centered research, which examines relationships between isolated variables across entire samples, this approach inherently overlooks the possibility that distinct subgroups of individuals may exist within the population. Variable-centered methods cannot capture how specific configurations of motivation and emotion co-occur within individuals to form holistic psychological profiles (Christie and Masyn, 2008; DeBusk-Lane et al., 2023).

A person-centered analytical technique, such as Latent Profile Analysis (LPA), is uniquely suited to address this limitation. LPA identifies unobserved subgroups, or profiles, based on individuals’ patterns of responses across multiple variables (Collier and Leite, 2017; Spurk et al., 2020; Tein et al., 2013). This is ideally suited for exercise psychology. Individuals do not possess a single, pure motivation like “intrinsic motivation.” Instead, they hold a combination of specific motives (e.g., enjoyment, appearance, fitness) and experience a mix of emotions during exercise (e.g., vitality, exhaustion). LPA can detect subgroups of students who share similar, multifaceted patterns of these specific motives and emotions. For example, one subgroup might be characterized by high fitness motives coupled with high enjoyment and vitality, while another might show high appearance motives alongside feelings of pressure and exhaustion.

Despite its utility, the application of LPA to the integrated study of exercise-specific motives and exercise-induced emotions is very limited. Some studies have used person-centered methods to profile either motivation or emotion in isolation (Zhao et al., 2025). However, few have combined these domains to examine how specific motivational contents and emotional responses naturally co-occur to form distinct psychological profiles. Furthermore, it is not well understood how demographic factors, such as gender and age, relate to membership in these integrated profiles. While gender differences in general motivation and activity levels are known, research has not tested whether gender predicts profile membership when profiles are defined by combinations of specific exercise motives and emotions.

Thus, there is a need for research that uses a person-centered approach (LPA) to identify subgroups based on the combined patterns of specific exercise motives and exercise-induced emotions, and to examine how key demographic variables like gender and age relate to these profiles.

### The current research

The present study aims to address gaps in existing research by using LPA to explore exercise motivation-emotion profiles and their links to physical activity among Chinese college students, with a focus on gender and age differences. Specifically, we address three objectives:

- Identify distinct latent profiles of college students based on their scores on specific exercise motives (enjoyment, competence, appearance, fitness, social) and exercise-induced emotions (vitality, tranquility, physical exhaustion, positive engagement);
- Examine whether gender and age predict membership in these latent profiles;
- Explore differences in physical activity levels across the identified profiles using methods that account for classification uncertainty.

By integrating motivation and emotions in a person-centered framework, this study seeks to provide a more nuanced understanding of how psychological factors shape physical activity in a non-Western college population, while offering insights for subgroup-specific exercise interventions.

## Methods

### Participants

This research recruited a total of 2,216 participants through convenience sampling. Blank questionnaires and those with identical responses across all items were excluded, resulting in a final sample of 1,586 college students. The sample had a mean age of 19.13 years with a standard deviation of 1.231, consisting of 468 males and 1,118 females. This sample size meets the minimum requirements for both structural equation modeling and latent profile analysis, as recommended in previous research (Xiong et al., 2015). For latent profile analysis specifically, the sample size is well above the commonly suggested threshold of 500 participants (Ferguson et al., 2020).

### Measures

#### Motivation for sport and exercise

The measurement of motivation in this study was rooted in Motives for Physical Activities Measure-Revised (MPAM-R) (Frederick and Ryan, 1993). The Chinese version of this scale consists of 15 items measuring five dimensions of exercise motivation: enjoyment, competence, appearance, fitness, and social. Participants responded to each item on a 7-point Likert scale from 1 (“Very inconsistent”) to 7 (“Very consistent”). In the current study, the internal consistency coefficients (*α*) for the five dimensions ranged from 0.76 to 0.84, indicating good reliability.

#### Exercise-induced feeling inventory

To assess participants’ global psychological responses to exercise, this study adopted the *Exercise-Induced Feeling Inventory (EFI)* (Gauvin and Rejeski, 1993). The EFI consists of 12 items divided into four dimensions: revitalization, tranquility, physical exhaustion, and positive engagement. Participants rated their current emotional states during or after exercise on a 5-point Likert scale from 1 (“Not at all”) to 5 (“Very strongly”). Internal consistency coefficients (α) ranging from 0.72 to 0.80 for the four dimensions.

#### Physical activity

Physical activity was measured with the International Physical Activity Questionnaire-Short Form (IPAQ-SF) (Craig et al., 2003). This widely validated tool is internationally recommended for assessing habitual physical activity and sedentary behavior in adults. It includes seven items. Six items ask about participation in vigorous-intensity activity, moderate-intensity activity and walking. One item assesses daily sedentary time such as sitting or reclining.

Following standard IPAQ scoring and previous research (Esmaeilzadeh et al., 2022), weekly physical activity volume was calculated in metabolic equivalent of task (MET)-minutes. The calculation combined weighted values for each activity type. Vigorous-intensity activity was weighted at 8.0 METs, moderate-intensity activity at 4.0 METs and walking at 3.3 METs. Each weight was multiplied by weekly frequency in days and daily duration in minutes before summing.

Prior to standardization and outlier removal, the raw MET-min/week scores for the sample were as follows (*M* = 2353.156, *SD* = 2513.349). For the current study, MET-minute values were standardized to meet statistical assumptions. Extreme univariate outliers (values beyond ±3.29 standard deviations) were identified using established criteria (Osborne, 2012) and excluded from subsequent parametric analyses to ensure the normality assumptions were not severely violated and to prevent these extreme values from exerting undue influence on the results.

#### Demographic variables

Participants provided basic demographic information including age, gender, and grade. For analysis, gender was coded as male = 1 and female = 2. In the subsequent multinomial logistic regression analysis, the higher coded value (female) served as the reference group for comparisons.

### Procedure

This study received approval from the Institutional Review Board of Sanming University before data collection (IRB: 2025ACNN-006). Data were collected from undergraduate students at a university in South China in September 2025. A total of 2,216 college students were recruited through *Wenjuanxin*, an online questionnaire platform commonly used in Chinese academic research.

Participants are adults so written informed consent was obtained directly from each person before they took part in the survey. Each participant completed the survey on their own and the process took 10 to 15 min. To improve the response rate and the quality of completed questionnaires, participants received a small incentive after submitting valid responses. Examples of incentives included stationery and coffee vouchers.

All data needed to replicate the study’s findings have been stored in the ScienceDB repository (https://doi.org/10.57760/sciencedb.31863). The data are publicly available for non-commercial academic research only. This follows open science practices and the reproducibility standards expected in empirical research.

### Statistical analysis

Following data collection, physical activity (PA) data were processed to calculate weekly metabolic equivalent of task (MET)-minutes. The standardized PA score was then examined for univariate outliers. Based on established conventions for continuous data (Osborne, 2012), cases with standardized scores beyond ±3.29 standard deviations were identified as extreme outliers. These 21 cases were excluded from the primary analysis to meet the assumptions of parametric tests and prevent undue influence on parameter estimates, resulting in a final analytic sample of 1,586 participants. To assess the robustness of this decision, a sensitivity analysis was conducted using a sample that retained these outliers (*n* = 1,607).

We used SPSS 25.0 for preliminary data screening and descriptive analyses. These analyses included calculating means, standard deviations and frequency distributions for all study variables. The software was also used to run Pearson correlation analyses, assess internal consistency reliability through Cronbach’s *α* coefficients and detect common method bias. For common method bias detection, we performed Harman’s single-factor test. All scale items were included in an unrotated exploratory factor analysis. A single factor explaining more than 40% of the total variance would indicate significant bias (Podsakoff et al., 2003).

Latent Profile Analysis (LPA) was conducted in Mplus 8.3 to identify subgroups based on the nine motivation and emotion indicators. Models with 1 to 6 profiles were estimated using maximum likelihood estimation with robust standard errors (MLR). To determine the optimal number of profiles, we considered multiple criteria: lower values of the Akaike Information Criterion (AIC), Bayesian Information Criterion (BIC), and sample-size adjusted BIC (aBIC); significant results of the Bootstrap Likelihood Ratio Test (BLRT) and the Lo–Mendell–Rubin Adjusted LRT (LMR); entropy values (higher indicating clearer classification); and the theoretical interpretability and practical utility of the profiles.

To account for classification uncertainty when examining predictors and outcomes, we employed the three-step method. Specifically, after establishing the unconditional LPA model, we used the R3STEP procedure to examine whether demographic covariates (gender, age) predicted latent profile membership. Additionally, we used the BCH method in a separate model to test for differences in the distal outcome (physical activity) across the latent profiles. This approach provides more accurate estimates than traditional two-step methods that ignore classification error.

*Step 1:* We estimated the unconditional latent profile model (as described above) using the nine indicator variables. The final three-profile solution was retained.

*Step 2*: We saved the probabilistic profile membership information (i.e., the posterior probabilities for each individual belonging to each profile) from the final unconditional model.

*Step 3 (Auxiliary Analysis):* We conducted separate auxiliary analyses using the saved classification information. First, to examine the effects of covariates (gender, age) on profile membership, we used the R3STEP command in Mplus. This procedure performs a multinomial logistic regression with the latent profile variable as the outcome, directly incorporating classification uncertainty. Second, to test for differences in the distal outcome (physical activity) across profiles, we used the Bolck-Croon-Hagenaars (BCH) method via the AUXILIARY (BCH) option in Mplus. The BCH method provides adjusted means and standard errors for the distal outcome within each profile and performs pairwise comparisons using Wald chi-square tests, all while correcting for the probability of profile misclassification.

These three-step procedures are superior to traditional two-step methods that rely on assigning individuals to their most likely profile, as they prevent bias from classification error.

## Results

### Common methods Bias

Common method bias is a type of systematic error. It happens when data for all study variables are collected through the same measurement method such as self-report questionnaires. This error can affect the true relationships between the constructs being studied. We used Harman’s single-factor test to check for potential common method bias (Podsakoff et al., 2003). We included all scale items in an unrotated exploratory factor analysis with principal component extraction and the results showed that the first factor explained 27.549% of the total variance. This percentage is below the commonly accepted critical level of 40%. The finding indicates that common method bias did not pose a serious problem for this study.

### Descriptive statistics and correlational analyses

Table 1 presents descriptive statistics and Pearson correlation coefficients for all study variables. The mean scores for exercise-induced emotion dimensions ranged from 2.88 (physical exhaustion) to 3.20 (positive engagement). For exercise motivation dimensions, mean scores varied between 3.61 (competence) and 4.06 (fitness). The mean age of participants was 19.13 years and physical activity scores had a mean of −0.075 after standardization.

**Table 1:** Descriptive statistics and correlations.

	Variables	*M*	*SD*	Ske.	Kur.	1	2	3	4	5	6	7	8	9	10	11
1	Revitalization	3.030	0.815	−0.005	0.446											
2	Tranquility	3.023	0.695	−0.129	0.783	0.608^***^										
3	Physical exhaustion	2.875	0.848	0.131	0.009	−0.387^***^	−0.349^***^									
4	Positive engagement	3.202	0.825	−0.105	0.336	0.852^***^	0.609^***^	−0.418^***^								
5	Social	3.794	0.727	−0.234	−0.239	0.317^***^	0.217^***^	−0.147^***^	0.351^***^							
6	Appearance	3.955	0.720	−0.365	−0.221	0.214^***^	0.111^***^	−0.026	0.190^***^	0.438^***^						
7	Competence	3.614	0.705	0.020	−0.283	0.429^***^	0.271^***^	−0.241^***^	0.452^***^	0.585^***^	0.448^***^					
8	Fitness	4.060	0.661	−0.343	−0.384	0.361^***^	0.221^***^	−0.192^***^	0.376^***^	0.555^***^	0.578^***^	0.608^***^				
9	Enjoyment	3.938	0.700	−0.337	−0.268	0.351^***^	0.219^***^	−0.162^***^	0.380^***^	0.697^***^	0.580^***^	0.632^***^	0.721^***^			
10	Physical activity	−0.075	0.710	1.753	3.473	0.215^***^	0.107^***^	−0.087^**^	0.221^***^	0.103^***^	0.051	0.263^***^	0.124^***^	0.109^***^		
11	Age	19.130	1.231	/	/	0.059^*^	0.078^**^	−0.134^***^	0.063^*^	0.002	−0.019	0.034	0.024	0.025	0.092^***^	
12	Gender	/	/	/	/	−0.156^***^	−0.118^***^	0.178^***^	−0.180^***^	−0.016	0.081^**^	−0.120^***^	−0.071^**^	−0.014	−0.332^***^	−0.162^***^

^*^p < 0.05, ^**^p < 0.01, ^***^p < 0.001. Ske, Skewness, Kur, Kurtosis.

Correlational analyses revealed consistent patterns aligned with prior studies. Positive exercise emotions showed significant positive correlations with motivation dimensions. Revitalization correlated strongly with positive engagement (*r* = 0.85) while both were positively associated with enjoyment (*r* = 0.35 and *r* = 0.38 respectively) and competence (*r* = 0.43 and *r* = 0.45 respectively). Physical exhaustion was negatively correlated with most motivation dimensions, including competence (*r* = −0.24) and fitness (*r* = −0.19).

Among motivation dimensions, enjoyment showed strong positive correlations with social (*r* = 0.70) and fitness (*r* = 0.72). Competence was closely linked to fitness (*r* = 0.61) and enjoyment (*r* = 0.63). Physical activity was positively associated with competence (*r* = 0.26) and revitalization (*r* = 0.22) but showed no significant correlation with appearance.

Gender was significantly correlated with several key variables. Males showed higher levels of revitalization (*r* = −0.16) and positive engagement (*r* = −0.18) while females reported higher physical exhaustion (*r* = 0.18) and appearance (*r* = 0.08). Age had weak but significant correlations with physical exhaustion (*r* = −0.13) and physical activity (*r* = 0.09).

All correlation coefficients with asterisks were statistically significant at the *p* < 0.05, *p* < 0.01 or *p* < 0.001 levels as indicated in Table 1. No extreme skewness or kurtosis values were observed, supporting the suitability of the data for subsequent parametric analyses.

### Latent profile analysis

Table 2 reports the information criteria, likelihood ratio test results and entropy values for latent profile models with 1 to 6 profiles (*n* = 1,586). We selected the optimal number of profiles based on multiple complementary criteria, as no single index can fully determine the best model.

**Table 2:** Latent profile model information criteria, likelihood ratio test, and entropy (*n* = 1,586).

Model	*K*	Log(*L*)	AIC	BIC	aBIC	Entropy	BLRT	LMR	Group size
1	18	−13784.817	27605.635	27702.276	27645.094	/	/	/	/
2	28	−12298.098	24652.196	24802.528	24713.577	0.789	<0.001	<0.001	0.489/0.511
3	38	−11781.592	23639.184	23843.204	23722.486	0.814	<0.001	0.021	0.147/0.506/0.347
4	48	−11442.273	22980.547	23238.257	23085.771	0.799	<0.001	<0.001	0.228/0.469/0.117/0.186
5	58	−11174.192	22464.383	22775.783	22591.529	0.812	0.004	<0.001	0.095/0.070/0.462/0.218/0.156
6	68	−10978.989	22093.979	22459.069	22243.047	0.811	<0.001	<0.001	0.080/0.211/0.363/0.066/0.178/0.102

**Figure 1:**
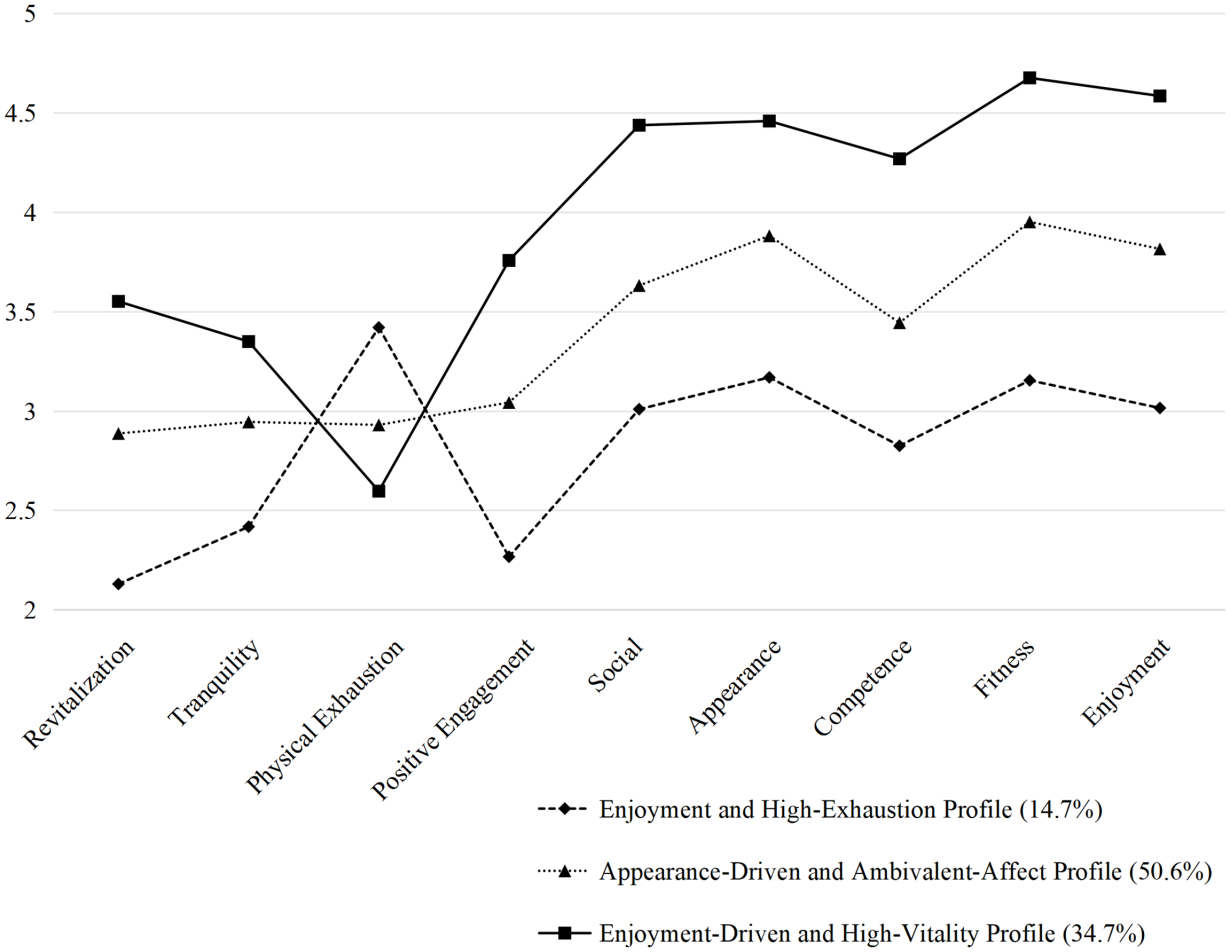
Motivational profiles of the best fitting model (3 profiles).

The selection of the final model was guided by a comprehensive evaluation of statistical evidence, classification certainty, theoretical interpretability, and practical utility. Although fit indices (AIC, BIC, aBIC) decreased with additional profiles, the magnitude of improvement diminished substantially beyond the three-profile solution, suggesting diminishing returns in model complexity for a marginal gain in fit. The three-profile solution demonstrated the highest entropy value (0.814), indicating superior classification accuracy compared to the four- (0.799) and five-profile (0.812) models. Crucially, upon examining the substantive meaning of alternative solutions, we found that models with more than three profiles primarily fragmented the large “Appearance-Driven and Ambivalent-Affect” profile into subgroups that differed quantitatively in motivation levels rather than presenting qualitatively distinct motivational-emotional patterns. Such subdivision offered no conceptually new insights and was indicative of statistical overfitting. Furthermore, the three-profile solution yielded well-sized, stable classes that are conceptually clear and actionable for tailoring interventions (i.e., maladaptive, conflicted, and adaptive groups). Balancing statistical parsimony, theoretical coherence, and practical relevance, the three-profile model was retained as optimal (as shown in Figure 1). Specifically, the additional profiles in the four- and five-class solutions represented mere splinters of the large ‘Appearance-Driven and Ambivalent-Affect’ profile, differing primarily in the degree rather than the qualitative pattern of motive-emotion combinations. A core tenet of person-centered analysis is to identify substantively distinct subgroups, not to create arbitrary distinctions along a continuum. The three-profile solution optimally balanced statistical fit with the principle of parsimony and identified subgroups that are theoretically meaningful and practically actionable for targeted interventions.

The final model comprised a Low-Enjoyment and High-Exhaustion Profile (*n* = 233, 14.7%) characterized by the lowest scores on the enjoyment and competence motive subscales and positive emotions coupled with the highest level of physical exhaustion. The Appearance-Driven and Ambivalent-Affect Profile (*n* = 803, 50.6%) represents the largest subgroup with moderate motivation levels and a blend of emotional experiences. The Enjoyment-Driven and High-Vitality Profile (*n* = 550, 34.8%) distinguished by the strongest the enjoyment, competence motive subscales and positive emotional states. The labels assigned to these three profiles are descriptive, derived directly from the salient characteristics of their respective MPAM-R and EFI score patterns. They serve as empirical summaries of the data. Interpretations regarding the alignment of these motive-affect patterns with broader motivational constructs from Self-Determination Theory (e.g., autonomous vs. controlled regulation) are provided in the Discussion as theoretical inferences, not as direct measurements.

### Associations between covariates and latent profile membership

The results of the three-step (R3STEP) multinomial logistic regression, which adjusts for classification uncertainty, are presented in Table 3. Using the Low-Enjoyment and High-Exhaustion Profile (Group 1) as the reference group, age did not significantly predict profile membership. The odds ratio for age was 1.085 [95% CI (0.902, 1.305), *p* = 0.379] when comparing the Enjoyment-Driven and High-Vitality Profile (Group 3) to the reference group, and 1.056 [95% CI (0.871, 1.280), *p* = 0.567] when comparing the Appearance-Driven and Ambivalent-Affect Profile (Group 2) to the reference group.

**Table 3:** Results of the three-step (R3STEP) multinomial logistic regression predicting latent profile membership.

Predictor	Profile comparison (vs. Profile 1)	Odds ratio (OR)	95% CI	*p*
Age	Profile 2 (Appearance-Driven and Ambivalent-Affect)	1.06	[0.87, 1.28]	0.567
	Profile 3 (Enjoyment-Driven and High-Vitality)	1.09	[0.90, 1.31]	0.379
Gender	Profile 2 (Appearance-Driven and Ambivalent-Affect)	1.24	[0.85, 1.81]	0.267
	Profile 3 (Enjoyment-Driven and High-Vitality)	2.15	[1.28, 3.62]	0.004

The reference group for profile comparison is Profile 1 (Low-Enjoyment and High-Exhaustion Profile). Gender was coded as 1 = Male, 2 = Female, with female serving as the reference group in the regression. An odds ratio greater than 1 indicates that male students have higher odds of belonging to the comparison profile than female students.

Gender significantly predicted profile membership. Using female students as the reference group, male students had significantly higher odds of belonging to the Enjoyment-Driven and High-Vitality Profile compared to the Low-Enjoyment and High-Exhaustion Profile [OR = 2.15, 95% CI (1.28, 3.62), *p* = 0.004]. Gender was not a significant predictor for membership in the Appearance-Driven and Ambivalent-Affect Profile relative to the same reference profile [OR = 1.24, 95% CI (0.85, 1.81), *p* = 0.267]. Age did not significantly predict profile membership.

### Differences in physical activity across latent profiles

Analyses were conducted to examine differences in physical activity levels based on demographic variables and latent profile membership. The results of a MANOVA, presented in Table 4, indicated a statistically significant main effect of gender on physical activity, with a small effect size (*F* = 9.566, *p* < 0.001, η^2^ = 0.015), indicating that male students reported significantly higher activity levels than female students. In contrast, no significant effect of age on physical activity was observed (*F* = 0.497, *p* = 0.353, η^2^ = 0.007).

**Table 4:** Results of MANOVA of age and gender.

Types	*F*	*p*	η^2^
Age	0.497	0.353	0.007
Gender	9.566	<0.001	0.015

Differences in physical activity (PA) levels across the three latent profiles were examined using the Bolck-Croon-Hagenaars (BCH) method, which adjusts for classification uncertainty. The BCH-adjusted mean PA levels (in standardized z-scores) for each profile are presented in Table 5. Contrary to the initial analysis that ignored classification error, the BCH results revealed a significant overall difference in PA across the three profiles, *χ*^2^(2) = 48.44, *p* < 0.001.

**Table 5:** Tests of physical activity differences across latent profiles using the BCH method.

Latent Profile	*n*	Adjusted mean (PA)	*SE*	Pairwise comparisons	*p*
1. Low-enjoyment and high-exhaustion	233	−0.218	0.050	/	/
2. Appearance-driven and ambivalent-affect	803	−0.184	0.025	vs. Profile 1: 0.331	0.565
3. Enjoyment-driven and high-vitality	550	0.131	0.039	vs. Profile 1: 30.525	<0.001
				vs. Profile 2: 40.518	<0.001

PA, Physical activity, presented as standardized scores. Means and standard errors (SE) are adjusted for classification uncertainty using the BCH method.

The Enjoyment-Driven and High-Vitality Profile (Group 3) demonstrated a significantly higher adjusted mean PA level (*M* = 0.13, *SE* = 0.04) compared to both the Low-Enjoyment and High-Exhaustion Profile (Group 1; *M* = −0.22, *SE* = 0.05; χ^2^(1) = 30.53, *p* < 0.001) and the Appearance-Driven and Ambivalent-Affect Profile (Group 2; *M* = −0.18, *SE* = 0.03; χ^2^(1) = 40.52, *p* < 0.001). No significant difference in PA was found between Group 1 and Group 2 (χ^2^(1) = 0.33, *p* = 0.565).

### Sensitivity analysis regarding outlier exclusion

A sensitivity analysis was conducted to assess the robustness of the primary findings to the exclusion of extreme outliers. This analysis included the 21 cases that were removed based on the ±3.29 standard deviation criterion for physical activity in the primary analysis, resulting in a sample of 1,607 participants.

The results were fully consistent with those of the primary analysis (as shown in Table 6). Firstly, latent profile analysis on this inclusive sample confirmed the same three-profile structure. Secondly, and most importantly, the pattern of physical activity differences across profiles remained unchanged. The Enjoyment-Driven and High-Vitality Profile demonstrated the highest adjusted mean physical activity level (*M* = 0.224, *SE* = 0.056) and was significantly higher than both the Low-Enjoyment and High-Exhaustion Profile (*χ*^2^ = 21.494, *p* < 0.001) and the Appearance-Driven and Ambivalent-Affect Profile (*χ*^2^ = 25.056, *p* < 0.001). Crucially, the comparison between the two lower-activity profiles remained non-significant (*χ*^2^ = 0.360, *p* = 0.548), closely mirroring the result from the primary analysis (*χ*^2^ = 0.33, *p* = 0.565).

**Table 6:** Comparison of physical activity differences across latent profiles in primary and sensitivity analyses.

Analysis	Sample size	Profile comparison	*χ* ^2^	*p*
Primary analysis	1,586	Profile 1 vs. Profile 2	0.33	0.565
		Profile 1 vs. Profile 3	30.53	<0.001
		Profile 2 vs. Profile 3	40.52	<0.001
Sensitivity analysis	1,607	Profile 1 vs. Profile 2	0.360	0.548
		Profile 1 vs. Profile 3	21.494	<0.001
		Profile 2 vs. Profile 3	25.056	<0.001

Profile 1 = Low-Enjoyment and High-Exhaustion; Profile 2 = Appearance-Driven and Ambivalent-Affect; Profile 3 = Enjoyment-Driven and High-Vitality.

This consistency across analytical approaches strengthens confidence in the main conclusions of the study. The identified motivation-emotion profiles are robust, and the specific pattern of physical activity engagement, in which only the most adaptive profile shows superior levels, is not an artifact of the decision to exclude extreme values.

## Discussion

This study employed latent profile analysis (LPA) to identify distinct subgroups of Chinese college students based on patterns of exercise motivation and exercise-induced emotions. The results revealed three psychologically meaningful profiles: a Low-Enjoyment and High-Exhaustion Profile, an Appearance-Driven and Ambivalent-Affect Profile, and an Enjoyment-Driven and High-Vitality Profile. Gender significantly predicted membership in the most adaptive profile, whereas age did not. Importantly, physical activity levels differed across these motivation-emotion profiles. Specifically, the Enjoyment-Driven and High-Vitality Profile demonstrated a significantly higher level of physical activity compared to the other two profiles, between which no significant difference was found. These findings demonstrate that the combination of autonomous motivation and positive affect is a hallmark of the most behaviorally engaged subgroup. The other two profiles, while both associated with lower activity levels compared to the adaptive profile, did not differ significantly from each other. This pattern, together with the significant role of gender, advances our theoretical understanding and offers clear pathways for practical application.

### Theoretical significance of the motivation-emotion profiles

The identified profiles substantiate the synergistic relationship between specific exercise motives and affective experiences, a premise central to SDT, which posits that the quality of motivation, fundamentally shapes behavioral and affective outcomes (Ryan and Deci, 2000). While the MPAM-R measures discrete motives rather than regulatory styles, its dimensions map conceptually onto this continuum. Motives such as enjoyment and competence are typically aligned with autonomous regulation, whereas appearance is often linked to more controlled forms of motivation (Teixeira et al., 2012).

Consequently, the Enjoyment-Driven and High-Vitality Profile embodies a high-quality, motivational pattern. The co-occurrence of strong intrinsic motives and the most positive emotional states (vitality, positive engagement) suggests that activities driven by inherent interest are more likely to satisfy basic psychological needs for autonomy and competence, thereby generating a positively reinforcing affective cycle (Ryan and Sapp, 2007; Standage et al., 2007).

In contrast, the Low-Enjoyment and High-Exhaustion Profile represents a maladaptive pattern akin to amotivation, where a pervasive lack of volition is coupled with the most negative affective outcome (Gunnell et al., 2013). The most prevalent Appearance-Driven and Ambivalent-Affect Profile offers a critical insight: exercising primarily for externally referenced reasons (e.g., body image) corresponds not to uniformly negative affect, but to psychological ambivalence—a mix of moderate positive and negative emotions. This aligns with SDT research indicating that controlled motivation yields less consistent affective benefits and may sustain engagement only while external contingencies are salient (Vansteenkiste et al., 2010). The dominance of this profile (50.6%) highlights a large student subgroup engaged in exercise but lacking the stable internal drive necessary for long-term adherence and well-being.

### Interpreting the pattern of physical activity differences

The analysis of physical activity outcomes revealed a specific differential pattern across the motivation-emotion profiles. Using the BCH method (Asparouhov and Muthén, 2014a, 2014b), which corrects for classification uncertainty, we found that the Enjoyment-Driven and High-Vitality Profile reported significantly higher PA levels than the other two profiles, which did not differ from each other. This finding refines our understanding of the motivation-emotion-behavior nexus. It demonstrates that superior behavioral engagement is uniquely associated with the synergistic presence of motives indicative of autonomy (i.e., high enjoyment and competence) and positive affect. Simply possessing some degree of motivation (as in the Appearance-Driven profile) is insufficient to elevate PA levels above those of the Low-Enjoyment and High-Exhaustion profile if that motivation is not accompanied by intrinsic enjoyment and vitality.

Several factors may explain this pattern. Firstly, powerful contextual constraints, such as the intense academic pressure and standardized schedules prevalent in Chinese universities, likely impose a ceiling on PA for most students (Jiang et al., 2024). Overcoming these barriers may require the robust internal resources characteristic of the most adaptive profile. Secondly, the global nature of the IPAQ-SF measure, while valid for estimating total volume (Craig et al., 2003), may obscure qualitative differences in how the two lower-activity profiles engage with exercise. Future research employing objective monitoring and assessing behavioral quality (e.g., consistency, enjoyment) could uncover further distinctions. Ultimately, this result underscores that the primary function of the motivation-affect pattern seen in the Enjoyment-Driven profile may be to enhance the quality, internalization, and long-term maintenance of behavior, with total volume being a more distal outcome under constrained environments (Williams et al., 2018).

### Associations between demographic variables and profile membership

Gender significantly predicted profile membership, with male students more likely to belong to the adaptive Enjoyment-Driven and High-Vitality Profile. This extends beyond simple activity-level disparities to reveal a gender-linked divergence in integrated psychological experiences of exercise. Socio-cultural factors, including greater encouragement for males in competitive and team sports, may provide more frequent opportunities to fulfill needs for competence and relatedness, thereby fostering intrinsic motivation (Chalabaev et al., 2013; Hermann and Vollmeyer, 2016). Conversely, the underrepresentation of female students in this optimal profile may reflect prevalent barriers such as a stronger societal emphasis on appearance-oriented goals (a controlled motive), lower perceived competence in traditional sports settings, and a relative lack of activities designed to support women’s autonomy and mastery (Boiché et al., 2014).

The absence of a significant age effect within this undergraduate cohort is an instructive finding. It suggests that the core structure of exercise motivation and emotion is relatively stable during this specific developmental period. This stability implies that the observed profiles are shaped more by enduring personal dispositions and social learning histories than by maturation alone during college years (Howard and Hoffman, 2018). Consequently, waiting for natural psychological maturation is an inadequate strategy; targeted interventions are necessary to facilitate shifts toward more adaptive engagement patterns.

### Robustness of findings and consideration of extreme values

To examine the robustness of the primary findings, a sensitivity analysis was conducted that included the 21 extreme outliers initially excluded based on the ±3.29 standard deviation criterion. The results were fully consistent with those from the primary analysis. The three-profile structure remained stable, and the specific pattern of physical activity differences was precisely replicated. Crucially, the comparison between the two lower-activity profiles remained non-significant, reaffirming the conclusion that they exhibit comparable engagement levels.

This consistency across analytical approaches confirms that the core motivational-emotional typology and its behavioral correlates are not artifacts of outlier management. It also highlights a methodological insight pertinent to person-centered research. While the latent structure itself proved highly robust, mean-level comparisons of continuous distal outcomes can be more susceptible to the influence of extreme values (Osborne, 2012). Our primary, conservative approach to outlier handling, which follows established recommendations (Leys et al., 2013), was therefore justified and yielded reliable estimates. Ultimately, the sensitivity analysis strengthens confidence in the main conclusion that superior physical activity is uniquely associated with the synergistic presence of autonomous motivation and positive affect.

### Practical implications for targeted interventions

The profile-based approach offers a precision framework for designing campus health promotions. Interventions should be tailored to the distinct needs of each subgroup:

For the Low-Enjoyment and High-Exhaustion Profile, the priority is to disrupt the negative association with exercise. Initiatives should focus on creating positive, low-pressure initial experiences that minimize performance evaluation. Socially oriented, non-competitive activities (e.g., recreational games, outdoor walks) can reduce feelings of exhaustion and gradually build a sense of competence and connectedness (Teixeira et al., 2012).

For the large Appearance-Driven and Ambivalent-Affect Profile, interventions should aim to facilitate motivational internalization. Combining factual health education with practical skill development can help students reframe exercise from an external obligation into a means for achieving personally valued goals like health, stress relief, and energy (Vansteenkiste et al., 2010). Mindfulness-based practices may also help manage appearance-related anxiety.

For the Enjoyment-Driven and High-Vitality Profile, the goal is maintenance and enrichment. Providing advanced skill workshops, leadership roles in campus clubs, or opportunities for organized competition can sustain their intrinsic motivation and allow them to serve as positive peer models (Standage et al., 2007).

Gender-sensitive programming is crucial. To better engage female students, universities should diversify offerings to include activities like yoga, dance, and self-defense, which may reduce appearance-related scrutiny and enhance autonomy. For all students, incentive systems should recognize participation and personal progress rather than performance outcomes, supporting a more autonomous climate (Fortier et al., 2007).

### Generalizability and future research directions

The generalizability of the identified motivation-emotion profiles warrants careful consideration. Certain aspects of these profiles may be particularly salient within the context of Chinese higher education. For instance, the prominence of the Appearance-Driven and Ambivalent-Affect Profile (50.6%) likely reflects the intersection of intense academic pressure, which can diminish opportunities for intrinsic enjoyment, and strong societal appearance-related norms (Hong et al., 2020; Jiang et al., 2024). Conversely, core psychological patterns delineated by the profiles have demonstrated relevance across diverse cultures (Teixeira et al., 2012).

Therefore, while the specific distribution and perhaps the salience of the “appearance-driven” pattern may be context-sensitive, the overarching typology of adaptive, conflicted, and maladaptive engagement is likely to be observable elsewhere, albeit with potential variations in prevalence and specific motive strength. Future research should directly test the cross-cultural replicability of these profiles using the same person-centered framework. This could involve multi-nation LPA studies or measurement invariance testing to determine if the profile structure holds across groups (Spurk et al., 2020). Additionally, applying this framework within different institutional settings (e.g., vocational colleges, universities with distinct sport policies) could reveal how environmental factors shape the emergence of these psychological patterns.

### Limitations and future research

This study has several limitations that should be noted. Firstly, it was limited to students from a single university in South China and was recruited via convenience sampling. Moreover, the sample’s gender distribution was notably imbalanced (468 males, 1,118 females). These characteristics may limit the generalizability of the results to other populations, such as students from different regions, more gender-balanced cohorts, or non-student groups. Future research should employ more representative and demographically balanced sampling strategies to test whether the identified latent profiles replicate across diverse populations.

Secondly, the cross-sectional design prevents us from exploring causal relationships between exercise motivation, emotion, and physical activity. We cannot be sure if profile membership leads to certain physical activity levels or if physical activity itself shapes motivation and emotion. Longitudinal studies that track participants over time would help clarify these causal links.

Thirdly, we did not include potential influencing factors like exercise type or social support. These variables might affect how motivation and emotion relate to profile membership and physical activity. Future work could add these factors to better understand the full range of influences on exercise behavior.

Fourthly, while latent profile analysis aims to identify qualitatively distinct subgroups, it is important to acknowledge that the underlying motivational and emotional constructs are continuous by nature. To some extent, our three profiles may represent salient regions along a continuum of exercise experience, rather than wholly discrete categories. This reflects a common consideration in person-centered methodology. Nevertheless, the observed differential associations of these profiles with key outcomes such as physical activity, as well as their prediction by demographic factors such as gender, support their practical utility and empirical validity as meaningful classifications. These profiles offer a useful, data-driven framework for understanding individual differences and for informing tailored intervention strategies.

Finally, the measure of physical activity relied on self-reported data, which may be affected by recall bias. Using objective tools such as activity trackers in future studies would provide more accurate physical activity data and strengthen the reliability of results.

## Conclusion

By employing a person-centered approach, this study delineated three distinct motivation-emotion profiles among Chinese college students, with the most adaptive profile being more common among males and uniquely associated with higher physical activity levels. The findings underscore that it is the confluence of motives centered on enjoyment and competence with positive affect that is critical for enhanced behavioral engagement. This nuanced understanding moves beyond variable-centered correlations to offer a typology that can directly inform the development of tailored, effective, and equitable interventions to promote physical activity and well-being on university campuses.

## Data Availability

The datasets presented in this study can be found in online repositories. The names of the repository/repositories and accession number(s) can be found in the article/Supplementary material.

## References

[ref1] Ashford S. Edmunds J. French D. P. (2010). What is the best way to change self-efficacy to promote lifestyle and recreational physical activity? A systematic review with meta-analysis. Br. J. Health Psychol. 15, 265–288. doi: 10.1348/135910709X461752, 19586583

[ref2] Asparouhov T. Muthén B. (2014a). Auxiliary variables in mixture modeling: three-step approaches using M plus. Mplus Web Notes 21, 329–341. doi: 10.1080/10705511.2014.915181

[ref3] Asparouhov T. Muthén B. (2014b). Auxiliary variables in mixture modeling: using the BCH method in mplus to estimate a distal outcome model and an arbitrary secondary model. Mplus Web Notes 21, 1–22.

[ref4] Boiché J. Plaza M. Chalabaev A. Guillet-Descas E. Sarrazin P. (2014). Social antecedents and consequences of gender-sport stereotypes during adolescence. Psychol. Women Q. 38, 259–274. doi: 10.1177/0361684313505844

[ref5] Buecker S. Simacek T. Ingwersen B. Terwiel S. Simonsmeier B. A. (2021). Physical activity and subjective well-being in healthy individuals: a meta-analytic review. Health Psychol. Rev. 15, 574–592. doi: 10.1080/17437199.2020.1760728, 32452716

[ref6] Castro O. Bennie J. Vergeer I. Bosselut G. Biddle S. J. (2018). Correlates of sedentary behaviour in university students: a systematic review. Prev. Med. 116, 194–202. doi: 10.1016/j.ypmed.2018.09.016, 30266213

[ref7] Castro O. Bennie J. Vergeer I. Bosselut G. Biddle S. J. (2020). How sedentary are university students? A systematic review and meta-analysis. Prev. Sci. 21, 332–343. doi: 10.1007/s11121-020-01093-8, 31975312

[ref8] Chalabaev A. Sarrazin P. Fontayne P. Boiché J. Clément-Guillotin C. (2013). The influence of sex stereotypes and gender roles on participation and performance in sport and exercise: review and future directions. Psychol. Sport Exerc. 14, 136–144. doi: 10.1016/j.psychsport.2012.10.005

[ref9] Christie C. A. Masyn K. E. (2008). Latent profiles of evaluators’ self-reported practices. Can. J. Program Eval. 23, 225–254. doi: 10.3138/cjpe.23.012

[ref10] Collier Z. K. Leite W. L. (2017). A comparison of three-step approaches for auxiliary variables in latent class and latent profile analysis. Struct. Equ. Model. 24, 819–830. doi: 10.1080/10705511.2017.1365304

[ref11] Craig C. L. Marshall A. L. Sjöström M. Bauman A. E. Booth M. L. Ainsworth B. E. . (2003). International physical activity questionnaire: 12-country reliability and validity. Med. Sci. Sports Exerc. 35, 1381–1395. doi: 10.1249/01.MSS.0000078924.61453.FB, 12900694

[ref12] DeBusk-Lane M. L. Zumbrunn S. Bae C. L. Broda M. D. Bruning R. Sjogren A. L. (2023). Variable- and person-centered approaches to examining construct-relevant multidimensionality in writing self-efficacy. Front. Psychol. 14:1091894. doi: 10.3389/fpsyg.2023.1091894, 36891200 PMC9986581

[ref13] Deci E. L. Ryan R. M. (2012). “Self-determination theory,” In Lange P. A. M. Van Kruglanski A. W. Higgins E. T. (eds) Handbook of theories of social psychology, 416–436 Thousand Oaks, CA: Sage

[ref14] Esmaeilzadeh S. Rodriquez-Negro J. Pesola A. J. (2022). A greater intrinsic, but not external, motivation toward physical activity is associated with a lower sitting time. Front. Psychol. 13:888758. doi: 10.3389/fpsyg.2022.888758, 35645933 PMC9133934

[ref15] Ferguson S. L. Moore E. W. G. Hull D. M. (2020). Finding latent groups in observed data: a primer on latent profile analysis in mplus for applied researchers. Int. J. Behav. Dev. 44, 458–468. doi: 10.1177/0165025419881721

[ref16] Fortier M. S. Sweet S. N. O’Sullivan T. L. Williams G. C. (2007). A self-determination process model of physical activity adoption in the context of a randomized controlled trial. Psychol. Sport Exerc. 8, 741–757. doi: 10.1016/j.psychsport.2006.10.006

[ref17] Frederick C. M. Ryan R. M. (1993). Differences in motivation for sport and exercise and their relations with participation and mental health. J. Sport Behav. 16, 124–146.

[ref18] Gauvin L. Rejeski W. J. (1993). The exercise-induced feeling inventory: development and initial validation. J. Sport Exerc. Psychol. 15, 403–423. doi: 10.1123/jsep.15.4.403

[ref19] Gillison F. B. Rouse P. Standage M. Sebire S. J. Ryan R. M. (2019). A meta-analysis of techniques to promote motivation for health behaviour change from a self-determination theory perspective. Health Psychol. Rev. 13, 110–130. doi: 10.1080/17437199.2018.1534071, 30295176

[ref20] Gunnell K. E. Crocker P. R. Wilson P. M. Mack D. E. Zumbo B. D. (2013). Psychological need satisfaction and thwarting: a test of basic psychological needs theory in physical activity contexts. Psychol. Sport Exerc. 14, 599–607. doi: 10.1016/j.psychsport.2013.03.007

[ref21] Hermann J. M. Vollmeyer R. (2016). “Girls should cook, rather than kick!”–female soccer players under stereotype threat. Psychol. Sport Exerc. 26, 94–101. doi: 10.1016/j.psychsport.2016.06.010

[ref22] Hong J.-T. Chen S.-T. Tang Y. Cao Z.-B. Zhuang J. Zhu Z. . (2020). Associations between various kinds of parental support and physical activity among children and adolescents in shanghai, China: gender and age differences. BMC Public Health 20:1161. doi: 10.1186/s12889-020-09254-8, 32711483 PMC7382138

[ref23] Howard M. C. Hoffman M. E. (2018). Variable-centered, person-centered, and person-specific approaches: where theory meets the method. Organ. Res. Methods 21, 846–876. doi: 10.1177/1094428117744021

[ref24] Jiang L. Cheskin L. J. Frankenfeld C. L. Rana Z. H. de Jonge L. (2024). Loneliness is associated with unhealthful dietary behaviors and physical inactivity among US college students. J. Am. Coll. Heal. 72, 2932–2937. doi: 10.1080/07448481.2022.2141060, 36395040

[ref25] Kwan M. Y. Cairney J. Faulkner G. E. Pullenayegum E. E. (2012). Physical activity and other health-risk behaviors during the transition into early adulthood: a longitudinal cohort study. Am. J. Prev. Med. 42, 14–20. doi: 10.1016/j.amepre.2011.08.026, 22176841

[ref26] Leys C. Ley C. Klein O. Bernard P. Licata L. (2013). Detecting outliers: do not use standard deviation around the mean, use absolute deviation around the median. J. Exp. Soc. Psychol. 49, 764–766. doi: 10.1016/j.jesp.2013.03.013

[ref27] Osborne J. W. (2012). Best practices in data cleaning: a complete guide to everything you need to do before and after collecting your data. Thousand Oaks, CA: Sage Publications.

[ref28] Pengpid S. Peltzer K. Kassean H. K. Tsala Tsala J. P. Sychareun V. Müller-Riemenschneider F. (2015). Physical inactivity and associated factors among university students in 23 low-, middle-and high-income countries. Int. J. Public Health 60, 539–549. doi: 10.1007/s00038-015-0680-0, 25926342

[ref29] Podsakoff P. M. MacKenzie S. B. Lee J.-Y. Podsakoff N. P. (2003). Common method biases in behavioral research: a critical review of the literature and recommended remedies. J. Appl. Psychol. 88:879. doi: 10.1037/0021-9010.88.5.879, 14516251

[ref30] Rhodes R. E. Kates A. (2015). Can the affective response to exercise predict future motives and physical activity behavior? A systematic review of published evidence. Ann. Behav. Med. 49, 715–731. doi: 10.1007/s12160-015-9704-5, 25921307

[ref31] Riemer B. A. Visio M. E. (2003). Gender typing of sports: an investigation of metheny’s classification. Res. Q. Exerc. Sport 74, 193–204. doi: 10.1080/02701367.2003.10609081, 12848232

[ref32] Rose E. A. Parfitt G. (2007). A quantitative analysis and qualitative explanation of the individual differences in affective responses to prescribed and self-selected exercise intensities. J. Sport Exerc. Psychol. 29, 281–309. doi: 10.1123/jsep.29.3.281, 17876968

[ref33] Ryan R. M. Deci E. L. (2000). Self-determination theory and the facilitation of intrinsic motivation, social development, and well-being. Am. Psychol. 55:68. doi: 10.1037//0003-066x.55.1.68, 11392867

[ref34] Ryan R. M. Sapp A. R. (2007). “Basic psychological needs: a self-determination theory perspective on the promotion of wellness across development and cultures” in Wellbeing in developing countries: from theory to research (Cambridge: Cambridge University Press), 71–92.

[ref35] Saemi E. Moteshareie E. Jalilinasab S. Afrash S. Deshayes M. (2023). Gender stereotypes and motor performance: how explicit and implicit stereotypes influence girls standing long jump and anxiety. Psychol. Sport Exerc. 64:102334. doi: 10.1016/j.psychsport.2022.102334, 37665817

[ref36] Spurk D. Hirschi A. Wang M. Valero D. Kauffeld S. (2020). Latent profile analysis: a review and “how to” guide of its application within vocational behavior research. J. Vocat. Behav. 120:103445. doi: 10.1016/j.jvb.2020.103445

[ref37] Standage M. Gillison F. Treasure D. C. (2007). “Self-determination and motivation in physical education” in Intrinsic motivation and self-determination in exercise and sport. eds. Hagger M. S. Chatzisarantis N. L. D. (Champaign, IL: Human Kinetics Publisher).

[ref38] Tein J.-Y. Coxe S. Cham H. (2013). Statistical power to detect the correct number of classes in latent profile analysis. Struct. Equ. Model. Multidiscip. J. 20, 640–657. doi: 10.1080/10705511.2013.824781, 24489457 PMC3904803

[ref39] Teixeira P. J. Carraça E. V. Markland D. Silva M. N. Ryan R. M. (2012). Exercise, physical activity, and self-determination theory: a systematic review. Int. J. Behav. Nutr. Phys. Act. 9:78. doi: 10.1186/1479-5868-9-78, 22726453 PMC3441783

[ref40] Uddin R. Burton N. W. Khan A. (2020). Combined effects of physical inactivity and sedentary behaviour on psychological distress among university-based young adults: a one-year prospective study. Psychiatry Q. 91, 191–202. doi: 10.1007/s11126-019-09697-2, 31811579

[ref41] Vansteenkiste M. Niemiec C. P. Soenens B. (2010). “The development of the five mini-theories of self-determination theory: an historical overview, emerging trends, and future directions” in The decade ahead: theoretical perspectives on motivation and achievement. eds. Urdan T. C. Karabenick S. A. (London: Emerald Group Publishing Limited), 105–165.

[ref42] Williams D. M. Rhodes R. Conner M. (eds). (2018). “Psychological hedonism, hedonic motivation, and health behavior” in Affective determinants of health behavior, (pp. 204–234). New York, NY: Oxford University Press.

[ref43] Xiong B. Skitmore M. Xia B. (2015). A critical review of structural equation modeling applications in construction research. Autom. Constr. 49, 59–70 (WOS:000347578800006). doi: 10.1016/j.autcon.2014.09.006

[ref44] Zhao Y. Wu Q. Zheng W. (2025). Motivation and physical activity across chinese adolescents: based on latent profile analysis. PLoS One 20:e0328383. doi: 10.1371/journal.pone.0328383, 40737243 PMC12310012

