# Supplementary material for: Latent profiles of exercise motivation and exercise-induced emotions: associations with physical activity and gender among Chinese college students
Source: Front Psychol. 2026 Mar 5;17:1759866. doi: 10.3389/fpsyg.2026.1759866 (PMC12999797; doi:10.3389/fpsyg.2026.1759866)
Supplement: Supplementary file 2 [file Table_2.docx]

Mplus VERSION 8.3

MUTHEN & MUTHEN

01/31/2026 2:25 PM

INPUT INSTRUCTIONS

TITLE: LPA with R3STEP for Covariates (Gender and Age);

DATA:

FILE = D:\Marco\analysis\Mplus\89\89_LPA.csv;

VARIABLE:

NAMES = age gender EFIHLJF EFISXPJ EFISLPB EFIJJTR MPAMSJ MPAMWM MPAMNL

MPAMJK MPAMLQ ZPA;

MISSING = ALL (999);

USEVARIABLE = EFIHLJF EFISXPJ EFISLPB EFIJJTR MPAMSJ MPAMWM MPAMNL

MPAMJK MPAMLQ;

CLASSES = c(3);

! 只使用R3STEP处理协变量

AUXILIARY = gender age (R3STEP);

ANALYSIS:

TYPE = MIXTURE;

STARTS = 500 100;

PROCESSORS = 4;

OUTPUT:

TECH11 TECH14;

SAVEDATA:

FILE = LPA_R3STEP_results.csv;

SAVE = CPROBABILITIES;

*** WARNING in MODEL command

All variables are uncorrelated with all other variables within class.

Check that this is what is intended.

1 WARNING(S) FOUND IN THE INPUT INSTRUCTIONS

LPA with R3STEP for Covariates (Gender and Age);

SUMMARY OF ANALYSIS

Number of groups 1

Number of observations 1586

Number of dependent variables 9

Number of independent variables 0

Number of continuous latent variables 0

Number of categorical latent variables 1

Observed dependent variables

Continuous

EFIHLJF EFISXPJ EFISLPB EFIJJTR MPAMSJ MPAMWM

MPAMNL MPAMJK MPAMLQ

Observed auxiliary variables

AGE

GENDER

Categorical latent variables

C

Estimator MLR

Information matrix OBSERVED

Optimization Specifications for the Quasi-Newton Algorithm for

Continuous Outcomes

Maximum number of iterations 100

Convergence criterion 0.100D-05

Optimization Specifications for the EM Algorithm

Maximum number of iterations 500

Convergence criteria

Loglikelihood change 0.100D-06

Relative loglikelihood change 0.100D-06

Derivative 0.100D-05

Optimization Specifications for the M step of the EM Algorithm for

Categorical Latent variables

Number of M step iterations 1

M step convergence criterion 0.100D-05

Basis for M step termination ITERATION

Optimization Specifications for the M step of the EM Algorithm for

Censored, Binary or Ordered Categorical (Ordinal), Unordered

Categorical (Nominal) and Count Outcomes

Number of M step iterations 1

M step convergence criterion 0.100D-05

Basis for M step termination ITERATION

Maximum value for logit thresholds 15

Minimum value for logit thresholds -15

Minimum expected cell size for chi-square 0.100D-01

Maximum number of iterations for H1 2000

Convergence criterion for H1 0.100D-03

Optimization algorithm EMA

Random Starts Specifications

Number of initial stage random starts 500

Number of final stage optimizations 100

Number of initial stage iterations 10

Initial stage convergence criterion 0.100D+01

Random starts scale 0.500D+01

Random seed for generating random starts 0

Input data file(s)

D:\Marco\analysis\Mplus\89\89_LPA.csv

Input data format FREE

SUMMARY OF DATA

Number of missing data patterns 3

Number of y missing data patterns 3

Number of u missing data patterns 0

COVARIANCE COVERAGE OF DATA

Minimum covariance coverage value 0.100

PROPORTION OF DATA PRESENT FOR Y

Covariance Coverage

EFIHLJF EFISXPJ EFISLPB EFIJJTR MPAMSJ

________ ________ ________ ________ ________

EFIHLJF 0.900

EFISXPJ 0.900 0.900

EFISLPB 0.900 0.900 0.900

EFIJJTR 0.900 0.900 0.900 0.900

MPAMSJ 0.736 0.736 0.736 0.736 0.837

MPAMWM 0.736 0.736 0.736 0.736 0.837

MPAMNL 0.736 0.736 0.736 0.736 0.837

MPAMJK 0.736 0.736 0.736 0.736 0.837

MPAMLQ 0.736 0.736 0.736 0.736 0.837

Covariance Coverage

MPAMWM MPAMNL MPAMJK MPAMLQ

________ ________ ________ ________

MPAMWM 0.837

MPAMNL 0.837 0.837

MPAMJK 0.837 0.837 0.837

MPAMLQ 0.837 0.837 0.837 0.837

UNIVARIATE SAMPLE STATISTICS

UNIVARIATE HIGHER-ORDER MOMENT DESCRIPTIVE STATISTICS

Variable/ Mean/ Skewness/ Minimum/ % with Percentiles

Sample Size Variance Kurtosis Maximum Min/Max 20%/60% 40%/80% Median

EFIHLJF 3.030 -0.005 1.000 2.87% 2.333 3.000 3.000

1427.000 0.664 0.440 5.000 3.22% 3.000 3.667

EFISXPJ 3.023 -0.129 1.000 1.47% 2.667 3.000 3.000

1427.000 0.483 0.776 5.000 1.33% 3.000 3.667

EFISLPB 2.875 0.131 1.000 2.94% 2.000 2.667 3.000

1427.000 0.718 0.005 5.000 2.38% 3.000 3.667

EFIJJTR 3.202 -0.104 1.000 2.17% 2.667 3.000 3.000

1427.000 0.681 0.330 5.000 4.63% 3.333 4.000

MPAMSJ 3.794 -0.234 1.333 0.23% 3.000 3.667 4.000

1327.000 0.528 -0.243 5.000 9.42% 4.000 4.333

MPAMWM 3.955 -0.365 1.333 0.08% 3.333 3.667 4.000

1327.000 0.518 -0.224 5.000 15.22% 4.000 4.667

MPAMNL 3.614 0.020 1.000 0.08% 3.000 3.333 3.667

1327.000 0.496 -0.287 5.000 5.73% 3.667 4.333

MPAMJK 4.060 -0.343 1.667 0.15% 3.667 4.000 4.000

1327.000 0.436 -0.387 5.000 16.58% 4.333 4.667

MPAMLQ 3.938 -0.337 1.667 0.15% 3.333 3.667 4.000

1327.000 0.448 -0.271 5.000 10.17% 4.000 4.667

RANDOM STARTS RESULTS RANKED FROM THE BEST TO THE WORST LOGLIKELIHOOD VALUES

Final stage loglikelihood values at local maxima, seeds, and initial stage start numbers:

-11781.592 985387 381

-11781.592 606576 151

-11781.592 349263 263

-11781.592 769907 457

-11781.592 17359 227

-11781.592 313407 132

-11781.592 40340 188

-11781.592 846194 93

-11781.592 153394 429

-11781.592 303634 169

-11781.592 366706 29

-11781.592 93468 3

-11781.592 458181 189

-11781.592 563002 360

-11781.592 765392 382

-11781.592 485256 371

-11781.592 937225 394

-11781.592 468036 131

-11781.592 496881 192

-11781.592 340112 126

-11781.592 418686 338

-11781.592 732596 320

-11781.592 715561 125

-11781.592 626891 32

-11781.592 22362 365

-11781.592 467339 66

-11781.592 193042 316

-11781.592 871851 257

-11781.592 507154 387

-11781.592 22089 143

-11781.592 30098 209

-11781.592 597614 284

-11781.592 488125 368

-11781.592 471438 252

-11781.592 53621 483

-11781.592 665121 417

-11781.592 259507 53

-11781.592 637345 19

-11781.592 937885 426

-11781.592 570908 98

-11781.592 253358 2

-11781.592 970689 266

-11781.592 840031 276

-11781.592 534864 307

-11781.592 217130 443

-11781.592 345070 114

-11781.592 814975 129

-11781.592 931874 141

-11781.592 529496 343

-11781.592 648555 113

-11781.592 21345 199

-11781.592 263221 447

-11781.592 170954 86

-11781.592 371246 101

-11781.592 68985 17

-11781.592 823392 479

-11781.592 887580 493

-11781.592 399848 220

-11781.592 973369 202

-11781.592 342019 301

-11781.592 568405 233

-11781.592 791285 416

-11781.592 246575 291

-11781.592 922596 456

-11781.592 227563 63

-11781.592 466971 109

-11781.592 404510 442

-11781.592 402224 91

-11781.592 891531 206

-11781.592 131856 90

-11781.592 314757 345

-11781.592 301180 236

-11781.592 673496 218

-11781.592 358488 264

-11781.592 443917 60

-11781.592 392418 28

-11781.592 749635 420

-11781.592 188498 258

-11781.592 392751 480

-11781.592 849670 347

-11781.592 791396 243

-11781.592 749453 33

-11781.592 281462 285

-11781.592 685657 69

-11781.592 762461 425

-11781.592 57226 208

-11781.592 347515 24

-11781.592 853195 431

-11781.592 21132 351

-11781.592 579995 183

-11781.592 195873 6

-11781.592 359578 458

-11781.592 281558 184

-11781.592 605161 409

-11781.592 997222 229

-11781.592 802682 419

-11781.592 521575 313

-11781.592 282464 283

-11781.592 395754 388

-11781.592 349360 464

THE BEST LOGLIKELIHOOD VALUE HAS BEEN REPLICATED. RERUN WITH AT LEAST TWICE THE

RANDOM STARTS TO CHECK THAT THE BEST LOGLIKELIHOOD IS STILL OBTAINED AND REPLICATED.

THE MODEL ESTIMATION TERMINATED NORMALLY

MODEL FIT INFORMATION

Number of Free Parameters 38

Loglikelihood

H0 Value -11781.592

H0 Scaling Correction Factor 1.4685

for MLR

Information Criteria

Akaike (AIC) 23639.184

Bayesian (BIC) 23843.204

Sample-Size Adjusted BIC 23722.486

(n* = (n + 2) / 24)

FINAL CLASS COUNTS AND PROPORTIONS FOR THE LATENT CLASSES

BASED ON THE ESTIMATED MODEL

Latent

Classes

1 240.51774 0.15165

2 786.86955 0.49613

3 558.61270 0.35221

FINAL CLASS COUNTS AND PROPORTIONS FOR THE LATENT CLASSES

BASED ON ESTIMATED POSTERIOR PROBABILITIES

Latent

Classes

1 240.51774 0.15165

2 786.86955 0.49613

3 558.61270 0.35221

FINAL CLASS COUNTS AND PROPORTIONS FOR THE LATENT CLASSES

BASED ON THEIR MOST LIKELY LATENT CLASS MEMBERSHIP

Class Counts and Proportions

Latent

Classes

1 233 0.14691

2 803 0.50631

3 550 0.34678

CLASSIFICATION QUALITY

Entropy 0.814

Average Latent Class Probabilities for Most Likely Latent Class Membership (Row)

by Latent Class (Column)

1 2 3

1 0.916 0.084 0.000

2 0.034 0.907 0.059

3 0.000 0.070 0.930

Classification Probabilities for the Most Likely Latent Class Membership (Column)

by Latent Class (Row)

1 2 3

1 0.888 0.112 0.000

2 0.025 0.926 0.049

3 0.000 0.085 0.915

Logits for the Classification Probabilities for the Most Likely Latent Class Membership (Column)

by Latent Class (Row)

1 2 3

1 8.542 6.474 0.000

2 -0.684 2.937 0.000

3 -10.016 -2.381 0.000

MODEL RESULTS

Two-Tailed

Estimate S.E. Est./S.E. P-Value

Latent Class 1

Means

EFIHLJF 2.131 0.202 10.535 0.000

EFISXPJ 2.419 0.162 14.969 0.000

EFISLPB 3.421 0.158 21.693 0.000

EFIJJTR 2.268 0.215 10.530 0.000

MPAMSJ 3.010 0.076 39.644 0.000

MPAMWM 3.170 0.071 44.874 0.000

MPAMNL 2.826 0.073 38.767 0.000

MPAMJK 3.155 0.058 53.945 0.000

MPAMLQ 3.016 0.070 42.947 0.000

Variances

EFIHLJF 0.446 0.030 15.044 0.000

EFISXPJ 0.392 0.020 19.509 0.000

EFISLPB 0.649 0.027 23.856 0.000

EFIJJTR 0.438 0.031 14.271 0.000

MPAMSJ 0.280 0.016 17.991 0.000

MPAMWM 0.334 0.018 18.778 0.000

MPAMNL 0.242 0.012 21.043 0.000

MPAMJK 0.175 0.017 10.372 0.000

MPAMLQ 0.167 0.014 12.298 0.000

Latent Class 2

Means

EFIHLJF 2.888 0.032 89.718 0.000

EFISXPJ 2.946 0.026 112.209 0.000

EFISLPB 2.931 0.032 91.615 0.000

EFIJJTR 3.044 0.031 97.392 0.000

MPAMSJ 3.632 0.049 73.828 0.000

MPAMWM 3.882 0.058 67.326 0.000

MPAMNL 3.445 0.048 71.465 0.000

MPAMJK 3.952 0.060 65.653 0.000

MPAMLQ 3.816 0.060 63.098 0.000

Variances

EFIHLJF 0.446 0.030 15.044 0.000

EFISXPJ 0.392 0.020 19.509 0.000

EFISLPB 0.649 0.027 23.856 0.000

EFIJJTR 0.438 0.031 14.271 0.000

MPAMSJ 0.280 0.016 17.991 0.000

MPAMWM 0.334 0.018 18.778 0.000

MPAMNL 0.242 0.012 21.043 0.000

MPAMJK 0.175 0.017 10.372 0.000

MPAMLQ 0.167 0.014 12.298 0.000

Latent Class 3

Means

EFIHLJF 3.552 0.055 64.776 0.000

EFISXPJ 3.350 0.040 83.262 0.000

EFISLPB 2.598 0.048 54.138 0.000

EFIJJTR 3.758 0.057 66.319 0.000

MPAMSJ 4.438 0.040 111.523 0.000

MPAMWM 4.459 0.031 143.201 0.000

MPAMNL 4.269 0.039 110.034 0.000

MPAMJK 4.676 0.032 147.990 0.000

MPAMLQ 4.585 0.030 153.775 0.000

Variances

EFIHLJF 0.446 0.030 15.044 0.000

EFISXPJ 0.392 0.020 19.509 0.000

EFISLPB 0.649 0.027 23.856 0.000

EFIJJTR 0.438 0.031 14.271 0.000

MPAMSJ 0.280 0.016 17.991 0.000

MPAMWM 0.334 0.018 18.778 0.000

MPAMNL 0.242 0.012 21.043 0.000

MPAMJK 0.175 0.017 10.372 0.000

MPAMLQ 0.167 0.014 12.298 0.000

Categorical Latent Variables

Means

C#1 -0.843 0.281 -2.995 0.003

C#2 0.343 0.084 4.062 0.000

QUALITY OF NUMERICAL RESULTS

Condition Number for the Information Matrix 0.232E-02

(ratio of smallest to largest eigenvalue)

TESTS OF CATEGORICAL LATENT VARIABLE MULTINOMIAL LOGISTIC REGRESSIONS USING

THE 3-STEP PROCEDURE

WARNING: LISTWISE DELETION IS APPLIED TO THE AUXILIARY VARIABLES IN THE

ANALYSIS. TO AVOID LISTWISE DELETION, DATA IMPUTATION CAN BE USED

FOR THE AUXILIARY VARIABLES FOLLOWED BY ANALYSIS WITH TYPE=IMPUTATION.

NUMBER OF DELETED OBSERVATIONS: 17

NUMBER OF OBSERVATIONS USED: 1569

Two-Tailed

Estimate S.E. Est./S.E. P-Value

C#1 ON

AGE -0.081 0.089 -0.915 0.360

C#2 ON

AGE -0.027 0.050 -0.535 0.593

Intercepts

C#1 0.714 1.693 0.422 0.673

C#2 0.850 0.961 0.884 0.377

Parameterization using Reference Class 1

C#2 ON

AGE 0.054 0.093 0.587 0.557

C#3 ON

AGE 0.081 0.089 0.915 0.360

Intercepts

C#2 0.135 1.767 0.077 0.939

C#3 -0.714 1.693 -0.422 0.673

Parameterization using Reference Class 2

C#1 ON

AGE -0.054 0.093 -0.587 0.557

C#3 ON

AGE 0.027 0.050 0.535 0.593

Intercepts

C#1 -0.135 1.767 -0.077 0.939

C#3 -0.850 0.961 -0.884 0.377

ODDS RATIOS FOR TESTS OF CATEGORICAL LATENT VARIABLE MULTINOMIAL LOGISTIC REGRESSIONS

USING THE 3-STEP PROCEDURE

(Est. - 1) Two-Tailed

Estimate S.E. / S.E. P-Value

C#1 ON

AGE 0.922 0.082 -0.954 0.340

C#2 ON

AGE 0.974 0.049 -0.542 0.588

Parameterization using Reference Class 1

C#2 ON

AGE 1.056 0.098 0.572 0.567

C#3 ON

AGE 1.085 0.096 0.879 0.379

Parameterization using Reference Class 2

C#1 ON

AGE 0.947 0.088 -0.604 0.546

C#3 ON

AGE 1.027 0.051 0.528 0.598

TECHNICAL 11 OUTPUT

Random Starts Specifications for the k-1 Class Analysis Model

Number of initial stage random starts 500

Number of final stage optimizations 100

VUONG-LO-MENDELL-RUBIN LIKELIHOOD RATIO TEST FOR 2 (H0) VERSUS 3 CLASSES

H0 Loglikelihood Value -12298.098

2 Times the Loglikelihood Difference 1033.013

Difference in the Number of Parameters 10

Mean 190.190

Standard Deviation 277.417

P-Value 0.0203

LO-MENDELL-RUBIN ADJUSTED LRT TEST

Value 1019.182

P-Value 0.0211

TECHNICAL 14 OUTPUT

Random Starts Specifications for the k-1 Class Analysis Model

Number of initial stage random starts 500

Number of final stage optimizations 100

Random Starts Specification for the k-1 Class Model for Generated Data

Number of initial stage random starts 0

Number of final stage optimizations for the

initial stage random starts 0

Random Starts Specification for the k Class Model for Generated Data

Number of initial stage random starts 40

Number of final stage optimizations 8

Number of bootstrap draws requested Varies

PARAMETRIC BOOTSTRAPPED LIKELIHOOD RATIO TEST FOR 2 (H0) VERSUS 3 CLASSES

H0 Loglikelihood Value -12298.098

2 Times the Loglikelihood Difference 1033.013

Difference in the Number of Parameters 10

Approximate P-Value 0.0000

Successful Bootstrap Draws 5

WARNING: OF THE 5 BOOTSTRAP DRAWS, 3 DRAWS HAD BOTH A SMALLER LRT VALUE THAN THE

OBSERVED LRT VALUE AND NOT A REPLICATED BEST LOGLIKELIHOOD VALUE FOR THE 3-CLASS MODEL.

THIS MEANS THAT THE P-VALUE MAY NOT BE TRUSTWORTHY DUE TO LOCAL MAXIMA.

INCREASE THE NUMBER OF RANDOM STARTS USING THE LRTSTARTS OPTION.

SAVEDATA INFORMATION

Save file

LPA_R3STEP_results.csv

Order and format of variables

EFIHLJF F10.3

EFISXPJ F10.3

EFISLPB F10.3

EFIJJTR F10.3

MPAMSJ F10.3

MPAMWM F10.3

MPAMNL F10.3

MPAMJK F10.3

MPAMLQ F10.3

AGE F10.3

GENDER F10.3

CPROB1 F10.3

CPROB2 F10.3

CPROB3 F10.3

C F10.3

Save file format

15F10.3

Save file record length 10000

DIAGRAM INFORMATION

Mplus diagrams are currently not available for Mixture analysis.

No diagram output was produced.

Beginning Time: 14:25:20

Ending Time: 14:25:54

Elapsed Time: 00:00:34

MUTHEN & MUTHEN

3463 Stoner Ave.

Los Angeles, CA 90066

Tel: (310) 391-9971

Fax: (310) 391-8971

Web: www.StatModel.com

Support: Support@StatModel.com

Copyright (c) 1998-2019 Muthen & Muthen
